# CB-Search: a method for searching for similar protein motifs with biased compositions

**DOI:** 10.1186/s12859-026-06509-w

**Published:** 2026-06-04

**Authors:** Patryk Jarnot

**Affiliations:** Department of Computer Networks and Systems, Silesian University of Technology, Akademicka 2A, 44-100 Gliwice, Poland

**Keywords:** Method, Similarity search, Protein sequence, Compositional bias

## Abstract

**Background:**

Methods for analyzing protein sequence similarity focus mainly on identifying the homologies of proteins with standard amino acid compositions. For motifs with biased compositions, they have already been shown to be suboptimal; thus, we lack dedicated tools to support their analyses. However, motifs with compositional biases also play key roles in protein functions. These domains can be found in transmembrane proteins, bind to RNA through RGG boxes, and may form prions. Nevertheless, many domains remain unknown, as for a long time they were considered nonfunctional and most of the methods mask them to improve homology searches. Therefore, we need better solutions to infer their functions more efficiently.

**Results:**

In this research, we developed a new method incorporating three alignment strategies, an algorithm for identifying motifs with similar compositions, 2-mer based filtering, and a new metric for evaluating alignments. These solutions focus mainly on comparing the physicochemical properties of protein sequences rather than their evolutionary relationships. To validate our approach, we compared BLAST with our method in three variants that use local, global–local, and global alignment with the algorithm for identifying compositionally similarities. We used these methods to search for similar transmembrane domains and RGG boxes. We observed that our solutions significantly increased the number of true positives. The greatest increase occurred after we applied our similarity score measure. Compositionally biased motifs frequently consist of two adjacent functionally important motifs; therefore, we also searched for similarities to the K-DE motif of DNA–directed RNA polymerase subunit delta. We found that compared with the other alignment strategies, global–local and global alignment with identifying similar regions included all the submotifs of the query sequence more often.

**Conclusion:**

Our method introduces novel strategies that enhance the search for compositionally biased motifs, thereby improving annotation retrieval via sequence matching.

## Background

Although protein similarity search algorithms are widely utilized to identify both homologous sequences and conserved motifs [1–3], their development has historically prioritized homology detection. Consequently, motif searches, particularly these with compositional biases, remain largely underaddressed. Scoring matrices, which are created from multiple sequence alignments of evolutionarily related proteins, are adjusted before each alignment to decrease the scores of frequently occurring residues to reduce the number of false positive hits introduced by low complexity regions [4]. Iterative methods such as PSI-BLAST and HMMER update multiple sequence alignments or Hidden Markov Model (HMM) profiles at each iteration to search for more distant homologies [5, 6]. The E-value is the standard metric for assessing alignment significance, representing the expected number of random alignments with a similar score in a target database. While this metric is essential for identifying evolutionarily related proteins, it becomes fundamentally unreliable when analyzing low-complexity regions due to the variable frequency of highly similar motifs [7, 8].

Protein fragments exhibiting such unusual sequence compositions can be categorized into four overlapping classes based on the degree to which specific residues dominate their composition and spatial arrangement: Compositionally Biased Regions (CBRs), Low-Complexity Regions (LCRs), Short Tandem Repeats (STRs), and homopolymers [9–15]. At the broadest level, CBRs encompass sequences where one or multiple residues are significantly overrepresented [12, 16, 17]. These biased residues may be distributed irregularly across the sequence, as seen in transmembrane domains, or organized into repeating structures like those found in collagen; they are typically identified using tools such as CAST and fLPS [10–12]. When a CBR is further defined by low informational entropy, it is designated as an LCR. LCRs can contain irregularly spaced residues or form STRs, and they are most commonly identified using the state-of-the-art method SEG [9]. STRs themselves consist of simple, repeated patterns that can either be highly conserved and visible or degenerate, comprising repetitions of physicochemically similar amino acids. Algorithms including SIMPLE, T-REKS, and XSTREAM are specifically designed to detect these repeat regions [14, 18, 19]. At the most restricted end of this spectrum are homopolymers, which are simply STRs formed by the repetition of a single residue type. The novel tool introduced in this paper focuses broadly on searching for motifs with similar biased compositions across this spectrum. Importantly, unlike identification methods that depend on absolute compositional thresholds, search methods must employ relative thresholds with respect to a query sequence to facilitate the search for similar motifs.

Search methods lack adequate support for similarity analyses of motifs with biased compositions, which however play many crucial roles in proteins. Among others, they can be found in regions that bind to RNA, transmembrane proteins and prions [20–22]. They are also frequently present in functional intrinsically disordered regions for which structure similarity based methods would fail [23]. These protein sequences unusual amino acid compositions are often created by different mechanisms than proteins with standard compositions. Homopolymers and other short tandem repeats are frequently formed through replication slippage [24]. DNA microsatellites, which encode these motifs, have a high mutation rate; thus, it is proposed that they form irregular low complexity regions in proteins [25–27]. To the best of our knowledge, the dominant evolutionary pathway for compositionally biased regions remains unclear. For instance, collagen is a large protein family of compositionally biased regions that evolved from a common ancestor. On the other hand, these proteins could have evolved independently in prokaryotes or migrated from eukaryotes through horizontal gene transfer [28, 29]. Consequently, solutions designed for homologous proteins with standard amino acid compositions have been already shown to be suboptimal for motifs with biased compositions [7, 30]. To the best of our knowledge, the literature offers only two previous attempts to solve this issue: one utilizing the Jaccard score and another involving a straightforward modification of BLAST parameters [7, 31]. It is important to acknowledge that scientists have reasonably focused first on homology detection of sequences with standard amino acid compositions because of their higher information content. However, motifs with biased compositions are now receiving increased attention, and we currently lack efficient tools for analyzing their similarities, which makes discovering their functions and structures challenging.

In this research, we developed a new method, called CB-Search, that is designed for searching for similar protein motifs that mostly relies on the physicochemical similarity of sequences with biased amino acid compositions rather than on homology. The method includes a new algorithm for identifying regions in proteins with similar compositions, implements three alignment strategies, 2–mer based filtering, and introduces a similarity score that is relevant for motifs with biased compositions.

## Materials and methods

The CB-Search method is designed for similar motifs with biased compositions. It offers three alignment strategies: global, local, and global–local. Needleman–Wunsch with gap affinity matrices as a global alignment algorithm can be used to align motifs previously identified by an external tool or by SimiComp, which identifies regions with similar amino acid compositions to a query sequence [32]. On the other hand, local and global–local alignments can find local similarities in an entire target sequence; thus, they can skip the identification step. As a local alignment, we implemented the Smith–Waterman algorithm with gap affinity matrices, whereas the global–local alignment is formally defined in the supplementary material in the section “Global–local alignment” [33]. The method also uses filtering that discards sequences lacking fragments with a given number of similar 2-mers, and utilizes a new similarity score metric. To evaluate our method, we searched for similar compositionally biased domains using CB-Search and BLAST [34]. The second method have been selected based on our prior work showing that even if BLAST have some flaws when searching for these domains, it performs better than CD-HIT and HHblits [7, 35, 36]. HHblits, while performing more sensitive homology searches than BLAST, cannot be used for motifs with biased compositions as these motifs may follow distinct evolution pathways. In particular, aligning two homopolymers of different lengths with a single mutation in one of the sequences, we cannot determine at which position in a profile of HMM this mutation should be accounted. We also tested MMseqs [37]. However, unsatisfactory results were obtained even when the recommended parameters were used for low complexity regions. For more information, see the Supplementary Material, section “Searching for compositionally biased motifs using MMseqs”.

### Global alignment of identified motifs

**Fig. 1:**
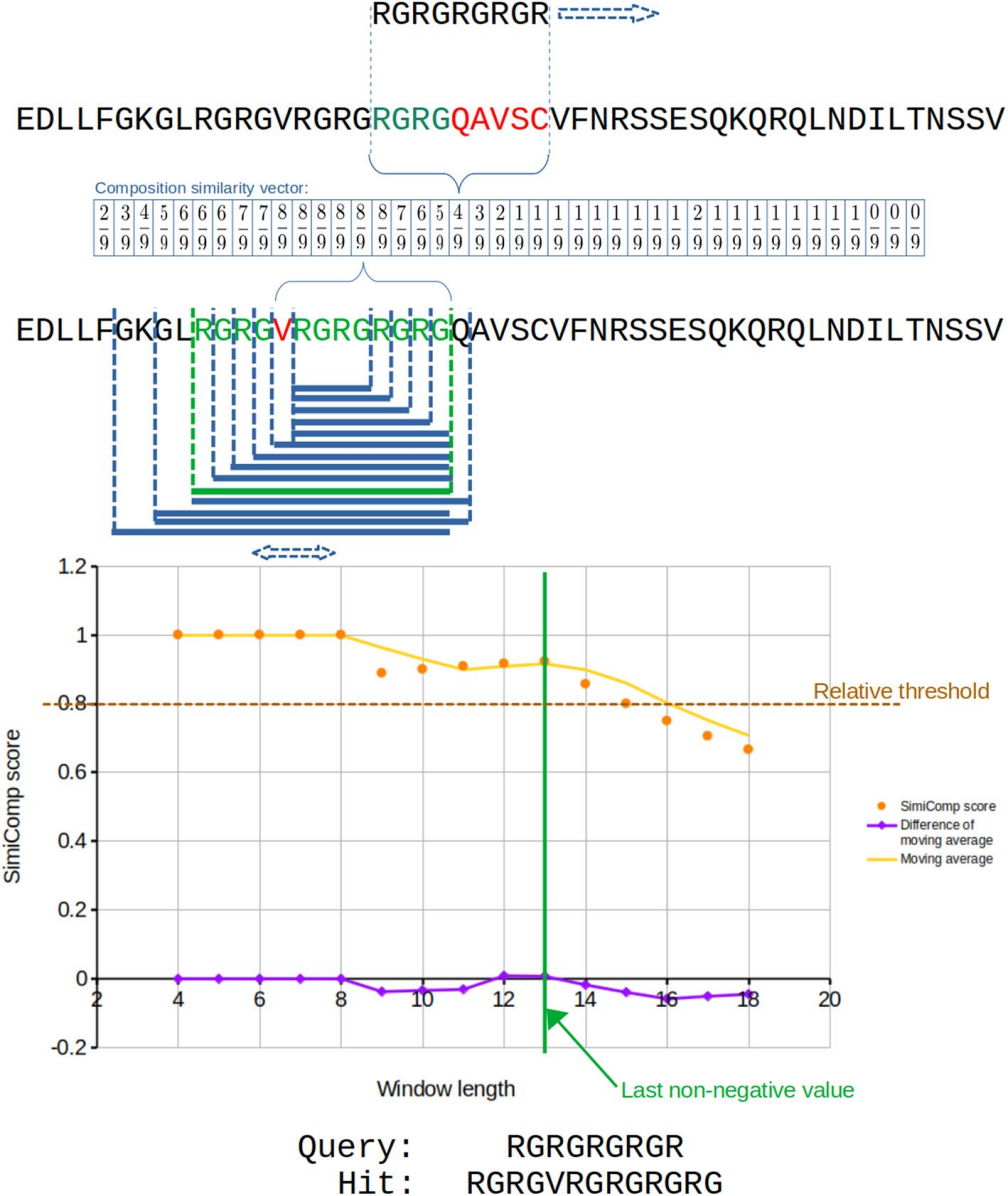
SimiComp first calculates a composition similarity vector and then identifies seed windows represented by local maxima in this vector. The next step involves shrinking and extending these regions. For each length, the method selects positions with the highest similarity scores. Finally, the similarity score is plotted against the window length, a simple moving average is applied, and the local maxima with the longest window length above the relative threshold are selected

SimiComp with the global alignment algorithm can identify motifs with similar compositions in database sequences, which are futher subjected to global alignment, applying gap penalties for differences in length. This method aims at finding fragments in database sequences that share similar amino acid composition with a query sequence. In its core, it uses a sliding window that is broadly utilized in dedicated tools identifying functional domains in protein sequences [38–40]. To select relevant regions, it utilizes relative composition threshold to a query sequence instead of absolute threshold as most of the identification methods. An example is shown in Fig. 1 where the method identifies a longer RG repeat region with a single insertion. Pseudocode of this algorithm is presented in Algorithm 1. Ultimately, by combining dynamic window sizing with a relative similarity threshold, SimiComp robustly identifies similar motifs and aligns them globally to the query, accurately accounting for length variations.

**Algorithm 1:**
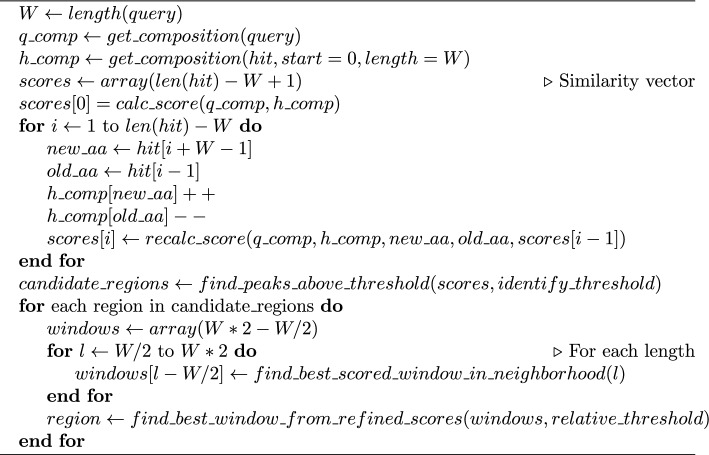
SimiComp

#### Amino acid frequency vector

It first calculates the amino acid frequency vector of a query sequence and then scans database sequences using a window size that matches the length of the query. Each window is then evaluated for similarity by calculating the frequency of common residues as in Equation 1.

1 $$\begin{aligned} c_k = \sum _{i=1}^N \min (p_i, p'_{ik}) \end{aligned}$$

where $p_i$ and $p'_{ik}$ represent the amino acid frequencies of a query and *k*-th window, respectively. *N* is the size of the alphabet.

#### Window length adjustment and evaluation

Given a frequency similarity vector *c*, the method determines intervals with values above a similarity threshold. In the next step, the method locates the maxima of these intervals, which become seeds of fragments with similar compositions to the query sequence. These seeds are further shrunk and expanded from 0.5*W* to 2*W* to determine the most relevant window size, where *W* is the query length. Each window size is used to scan the seed window within the boundaries *<B, E>*, as defined by Eqs. 2 and 3.

2 $$\begin{aligned}&B = \max (\min (b, e - l), 0)\end{aligned}$$

3 $$\begin{aligned}&E = \min (\max (e, b + l), L) \end{aligned}$$

where *b* is the beginning position of the initial window, *e* is the ending position of this window, *l* is the window length, and *L* is the database sequence length. As a result, we obtain sequence boundaries within which we calculate the similarity vector using Eq. 1, and for each window length, we select the window with the highest score.

#### Best window selection

Having windows with maximal scores for each length, we aim to choose a window that represents a balance between length and similarity, allowing for some mutations in the final regions to include more relevant fragments. For instance, when comparing an exact poly-Q region with a longer region containing some mutations, our goal is to label the entire degenerated poly-Q as sequence similarity. On the other hand, we aim to avoid extending regions because of random relevant residues. Therefore, the algorithm extends regions as long as they contain significant segments of relevant residues. To identify these segments, the method involves constructing a vector *v* of maximal composition similarity scores for each window length using Eq. 1, which are then sorted in ascending order. It then applies a simple moving average to this vector to filter noise from random matches. In this vector, the method selects a local maxima with the highest window length and a value above *\max (v)-r*, where *r* is the relative threshold.

#### Post-identification evaluation

This method identifies fragments with similar compositions, but residues may be arranged differently in both sequences. To evaluate the similarity of these sequences in terms of both composition and arrangement, we next align them globally using Needleman-Wunsch. This global alignment algorithm additionally penalizes differences in the lengths of the compared sequences.

### 2-mer filtering

This prealignment filtering excludes from analysis sequences lacking fragments that share at least a specified fraction of identical 2-mers with a query sequence. The utilization of such overlapping substrings, broadly referred to as *k*-mers, is a highly common strategy employed across numerous bioinformatics tools for efficient sequence comparison and heuristic filtering [35, 41, 42]. Compositionally biased fragments of proteins, due to overrepresentance of a given residue types, frequently contain repetitions of short patterns. The idea of analysing repeating seeds is frequently used in methods designed for degenerated tandem repeat detection, and here we use it also to sequences in which these repeats occur in inrregular manner for filtering out likely irrelevant sequences [18, 19]. The method begins by counting all 2-mers in the query sequence and then scans the target sequence using a window length that matches the length of the query sequence. In each window, it counts 2-mers and compares them to those from the query. If the fraction of identical 2-mers is greater than or equal to a specified threshold, the target sequence is considered to potentially contain a similar protein sequence and is subjected to further alignment. Please note that all 2-mers must be counted for the first window only. For subsequent windows, the method removes the leftmost 2-mer from the previous window and adds the rightmost 2-mer from the new window. Pseudocode of this procedure is presented in Algorithm 2.

**Algorithm 2:**
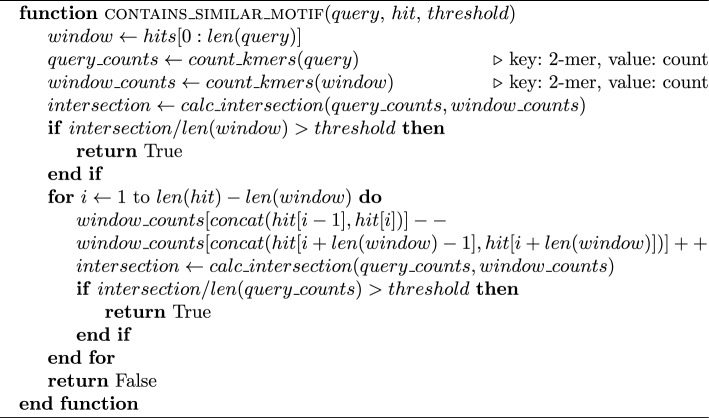
2-mer filtering

### Alignment similarity

The similarity score introduced in CB-Search is simple yet effective. It (1) makes results calculated using different scoring matrices and databases comparable, (2) solves the issue of evaluating alignments computed using scoring matrices with positive expected scores, and (3) enables the evaluation of the similarity between low complexity regions [43]. This score is described by Eq. 4:

4 $$\begin{aligned} S'' = \frac{s}{q} \end{aligned}$$

where *s* is the raw score of an alignment and *q* is the raw score of a query sequence self-alignment. For positive gap penalties, all match scores are greater than or equal to the mismatch scores in a scoring matrix $S'' \in (-\infty , 1]$. Additionally, *q* can be computed by summing all the match scores of a query sequence’s residues, as formally described in Eq. 5:

5 $$\begin{aligned} q = \sum _{i=1}^{L}s(x_i, x_i) \end{aligned}$$

where *L* is the length of the query sequence and $x_i$ represents the amino acids in this sequence.

Our similarity score normalizes the scores derived from frequency-based scoring matrices calculated with different logarithmic bases. For an ungapped alignment, the normalized score, which utilizes the logarithm of the target-to-background frequency ratio, can be calculated using Eq. 6:

6 $$\begin{aligned} S'' = \frac{\sum _{i=1}^{L} \log _c(f_i)}{\sum _{j=1}^{L} \log _c(g_j)} \end{aligned}$$

where *L* is the length of the alignment, $f_i$ is the target-to-background frequency ratio for the alignment at position *i*, and $g_j$ is the corresponding frequency ratio for a self-alignment at position *j*. Please note that, according to the logarithmic change-of-base rule, the final result is independent of the intermediate base *c*. This eliminates the need to calculate the *λ * parameter, as is required in BLAST and therefore allows for using scoring matrices with positive expected score [43].

### Similarity analysis of transmembrane regions

To validate our solutions, we compared three parameter settings of CB-Search with those of BLAST in the similarity analysis of transmembrane domains within the UniProtKB/Swiss-Prot database version 2023_03 [44]. To find these parameter settings, we optimized the gap penalties for the three available alignment algorithms using grid search. The first configuration, referred to as CB-Search-LC, employed local alignment; the second configuration, CB-Search-GL, employed global–local alignment; and the third configuration, called CB-Search-SC, utilized SimiComp with global alignment. The optimization algorithm, searches for the optimal solution with gap opening penalties ranging from 6 to 13 in increments of 1 and gap extension penalties ranging from 0.5 to 2 in increments of 0.5 for CB-Search. BLAST, however, allows for only predefined gap penalty pairs for BLOSUM62, which are: (11, 2), (10, 2), (9, 2), (8, 2), (7, 2), (6, 2), (13, 1), (12, 1), (11, 1), (10, 1), and (9, 1) for gap opening and gap extension penalties, respectively. This limitation of BLAST justifies the selection of CB-Search threshold ranges. To analyze the low complexity domains only, we utilized all the motifs from LCRAnnotationsDB that overlap at least 80% with LCRs identified with strict parameter (locut: 1.5, hicut: 1.8, window: 15) settings and contain the ’transmembrane’ keyword in their annotations. Following these criteria, we obtained 248 queries. As a positive set, we included all proteins that have the keyword ’transmembrane’ in their annotations, as some closely related sequences may be outside the SEG-strict threshold [45]. It contained 56,574 records. For the optimization, we maximized the number of true positives in the top 50 results of all the queries. It is worth noting that these annotations, while derived from well–curated Swiss–Prot subset of UniProtKB sequences, may still contain automatically annotated proteins. Nevertheless, when comparing search tools, all of them are equally affected by this noise [46]. This is a common issue with biological data; while some benchmark datasets are created for critical assessment of new methods, such a solution is not currently available for motifs with biased compositions [47, 48].

Next, to compare the selected alignment algorithms, we employed the optimal parameters when searching for similarities. For this analysis, we used the limit of top hits as a threshold of 50 times ranging from 1 to 50. This threshold allows us to compare alignment algorithms used in CB-Search, as true positives in the top results determine whether the results are ordered for each query by relevance.

To evaluate all CB-Search features against BLAST, we also searched for top similarities using the similarity score and E-value metrics as thresholds. For each method, we first established boundary thresholds, synchronizing them on the basis of hit counts. We determined the starting point by searching for a threshold yielding approximately 248 hits, which is equal to the number of queries, whereas the ending point was aimed at thresholds that yielded 20 times more hits. We used a binary search with 15 comparisons to determine these intervals. Next, for each method we searched for similarities 20 times by changing the thresholds from the starting point to the ending point, with uniform intervals. For CB-Search, we used the similarity score as the metric, whereas for BLAST, we utilized the E-value, and adjusted *x*, denoted as $10^x$. For CB-Search-SC, we searched from 0.58 to 0.88; for CB-Search-GL from 0.66 to 0.91; for CB-Search-LC, from 0.61 to 0.91; and for BLAST, we used *x* values ranging from −3.86 to −0.20.

Finally, we also analysed the impact of 2-mer filtering on true positive content in search results. Transmembrane regions may be found in nonhomologous proteins; thus, they may be considered challenging for such solutions. We used all the CB-Search variants to search for similar domains using optimised parameters and 2-mer filter in range *<0.0, 0.6>*. Using this data, we ploted a chart of true to false positive fraction and 2-mer filter threshold. The searches were performed on Intel(R) Xeon(R) CPU E5-4667 v4 @ 2.20 GHz, 72 cores, 144 threads.

### Similarity analysis of RGG boxes

We also applied the analysis of similar domains to RGG boxes to reinforce our conclusions. Here, however, we employed compositional bias criteria for the input dataset. As queries, we utilized regions from UniProtKB/Swiss-Prot containing the ’RGG’ keyword in the notes section and exhibiting more than 30% bias toward at least one residue. This resulted in 114 regions found in 87 proteins, whereas there were 107 proteins containing the ’RGG’ keyword in total. The last criterion was omitted when the hits were classified as true and false positives. In this analysis, we also optimized the alignment parameters using the top 50 hits and evaluated the performance of the alignment algorithms by setting the limit of the top results as the threshold. Finally, we used the similarity score and e–value as thresholds for CB-Search and BLAST, respectively. To maintain a comparable number of results, we searched for threshold values for each method using binary search, yielding approximately 114 and 2,280 hits. As a result, for CB-Search-SC, we applied a similarity score with 20 values ranging from 0.61 to 0.98, whereas for CB-Search-GL and CB-Search-LC, we applied values from 0.72 to 0.99. BLAST employs the e–value as a metric to assess the relevance of a target sequence, and we use *x*, denoted as $10^x$, which ranges from −25.97 to −11.67.

### Similarity analysis of keratin type II head

For the third case study, we analyzed the keratin type II head domain, with sequence data retrieved from the InterPro database [49]. Consistent with our methodology for RGG boxes, we utilized compositional bias criteria to curate the input dataset, selecting 27 sequences from the 90 available keratin type II head domains to serve as queries. Alignment parameters were optimized using the top 50 hits, and algorithm performance was subsequently evaluated by establishing the top results as the classification threshold. To generate the curves of true to false positive ratio, we applied specific similarity score ranges for each tool: 0.19 to 0.62 for CB-Search-SC, 0.25 to 0.66 for CB-Search-GL, and 0.24 to 0.66 for CB-Search-LC. For BLAST, the E-value threshold was varied using $10^x$, where *x* ranged from −57.43 to −18.95.

### Example: K/DE motif search

In this example, we utilized CB-Search and BLAST to search for similarities to the K-DE motif of DNA-directed RNA polymerase subunit delta (P12464) within the UniProtKB/Swiss-Prot database [44]. This motif, located at position 98–171, contains 9 lysines at the N-terminus and 48 aspartic/glutamic acids in the middle and at the C-terminus It has been shown that the lysines located and the N-terminus of this motif, have a significant effect on transcription activity [50]. However, we expected that local alignment would favor more accurate alignments to the DE submotif than to the whole but slightly less accurate K-DE motifs. To evaluate the results, we calculated the number of lysine matches in each midline of the alignments. For CB-Search, we optimized gap opening and gap extension penalties ranging from 6 to 13 with step 1, and from 0.5 to 2 with step 0.5, respectively for all available alignment algorithms. We also optimized similarity and relative thresholds where applicable. For the similarity threshold, we tested values of 0.65, 0.7, and 0.75, whereas for the relative threshold, we checked values of 0.1, 0.05, and 0.01. In the case of BLAST, we used the available combinations of gap penalties and word size. Finally, we compared the results using the parameters that yielded the greatest number of matched lysines.

To evaluate the scalability of our method and determine how execution time scales along with increasing dataset size, we conducted performance testing across ten datasets of progressively varying sizes. The initial baseline dataset consisted entirely of the UniProtKB/Swiss-Prot sequences. To construct the subsequent databases, we iteratively appended randomly sampled sequences (without replacement), with each newly added chunk being exactly equal in size, in terms of sequence count, to the original UniProtKB/Swiss-Prot database. For each of the ten resulting databases, similarity searches were executed ten independent searches, and the corresponding execution times were recorded to ensure reliability. For searches, we used optimised parameters found for the K-DE motif. Finally, the relationship between the total number of database sequences and the search execution time was plotted to visualize the method’s time complexity.

## Results

In this section, we present the results of the biological analyses comparing CB-Search with BLAST. We utilized CB-Search in three variants: CB-Search-LC which employs local alignment algorithm, CB-Search-GL which uses global–local alignment, and CB-Search-SC, which uses SimiComp with global alignment. First, we compared the methods for searching for similar transmembrane domains that overlap with the SEG method and RGG boxes, which exhibit a minimum 30% bias toward at least one residue. Afterward, we applied methods to search for similarities to the K-DE motif of DNA-directed RNA polymerase subunit delta.

### Analysis of the similarity of transmembrane domains, RGG boxes, and keratin type II head

**Fig. 2:**
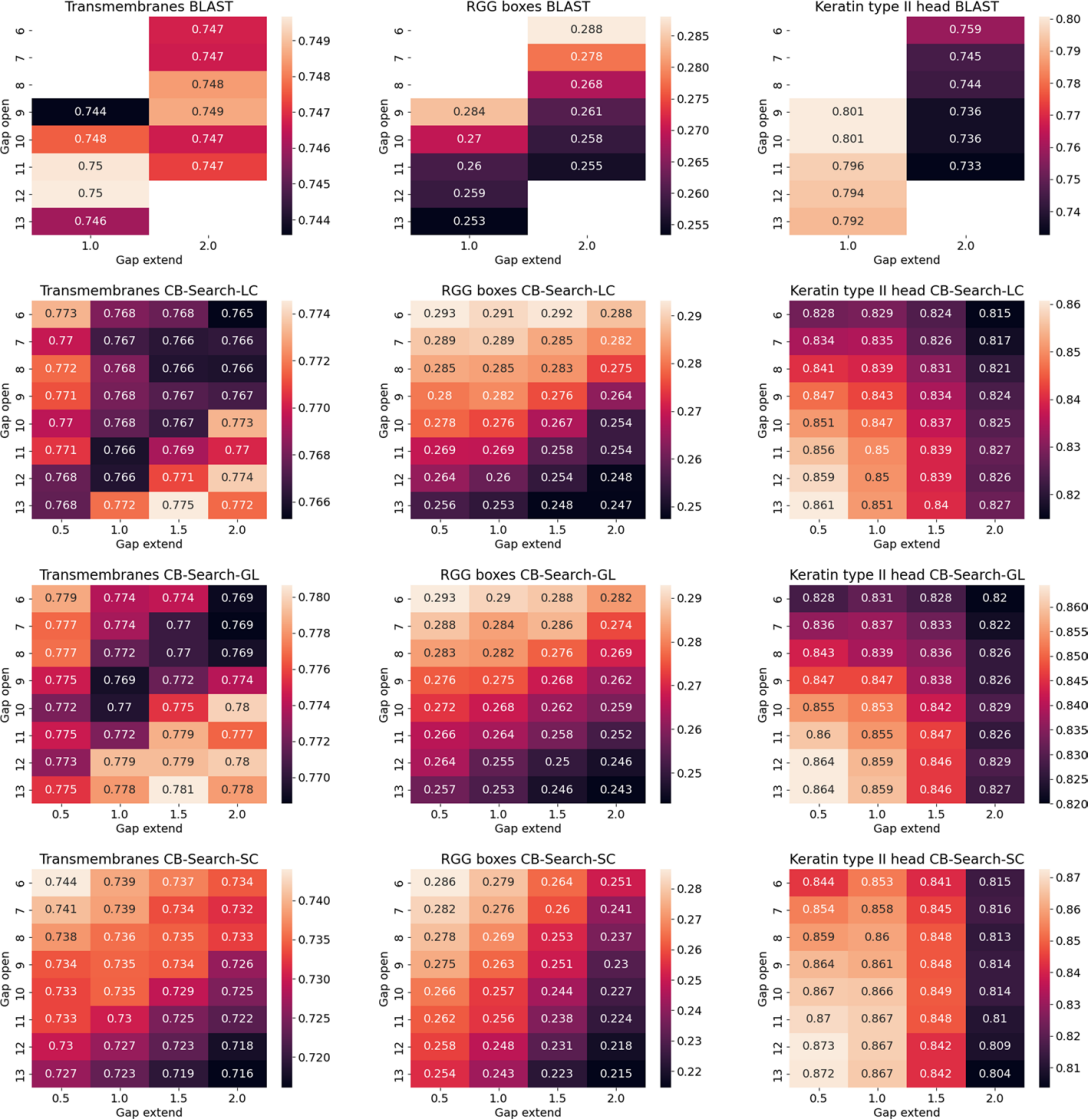
Compared with the other methods, parameter optimization had a greater effect on CB-Search-SC. For CB-Search-SC, the fraction of true positives clearly increased as the penalty decreased, with opposite tendency for keratin. This trend is also observable for CB-Search-GL and CB-Search-LC, but only in the case of RGG boxes and keratin. The heatmap illustrates the optimization results of the gap opening and gap extension penalties in relation to the fraction of true positives

In the analyses of the transmembrane domains, RGG boxes, and keratin type II head, we first adjusted the alignment parameters to evaluate their impact on the fraction of true positives in the results, and to select the optimal parameter sets for further comparison. As shown in Fig. 2, optimization had a slightly greater effect on CB-Search-SC than on the other methods, even when only the values available in BLAST were considered. For transmembranes, the difference between the maximal and minimal values was 0.01 for CB-Search-LC, 0.012 for CB-Search-GL and 0.028 for CB-Search-SC, indicating that the variation for CB-Search-SC was more than twice that of CB-Search-GL and CB-Search-LC. Additionally, the results from CB-Search-SC demonstrated a nearly consistent increase in the fraction of true positives as the gap opening penalty decreased, suggesting that further reductions in this parameter could enhance the results. In terms of the fraction of true positives for the limit of 50 hits, CB-Search-GL slightly outperforms the other alignment strategies. However, for RGG boxes, the difference was minimal, and this outcome could differ if BLAST permitted a wider range of gap penalty values. In the case of keratin’s domain, the gap open parameter was optimised towards the opposite direction, and more true positives were found when increasing its value. Utilization of the default CB-Search parameters (gap opening penalty of 10, gap extension penalty of 0.5) resulted in an average deviation from optimal values of 1.1%, with a standard deviation of 0.6% and a range of 0.5% to 2.1%. Consequently, to achieve peak performance, it is necessary to optimize these parameters using available annotations prior to subsequent analyses.

**Fig. 3:**
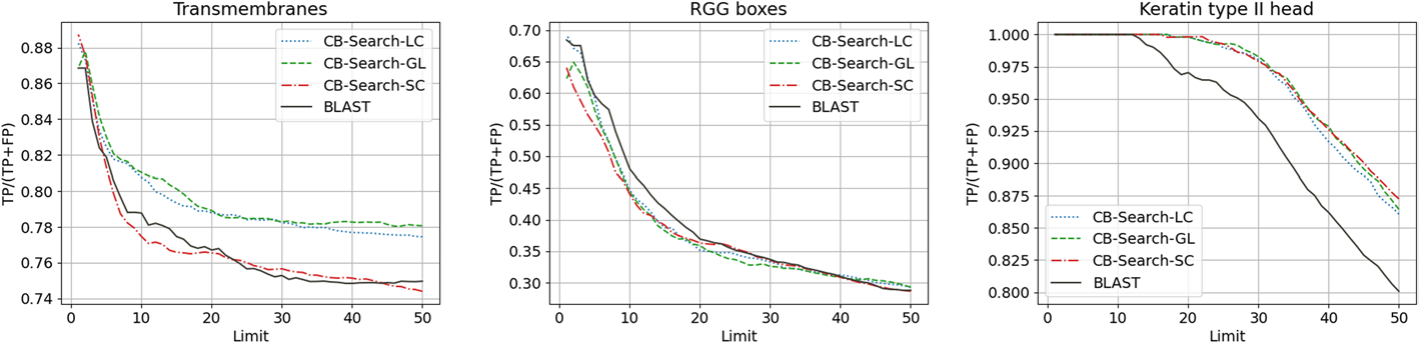
Compared with the other methods, CB-Search-GL and CB-Search-LC resulted in more true positives when more distant similarities in transmembrane domains were identified. In contrast, BLAST revealed more true positives for RGG boxes with closer similarity. For keratin, all CB-search variants found more true positives than BLSAT. The charts present fractions of true positives by the number of hits per query. Please note the difference in scales between the charts

The comparison of the alignment algorithms did not reveal significant or consistent differences in terms of the number of true positives among the selected alignment algorithms. In Fig. 3a, starting from *k=25*, CB-Search-LC and CB-Search-GL compared with other methods, yielded approximately 3% more true positives, while the results from BLAST and CB-Search-SC varied closely. In contrast, the results for RGG boxes differed, with BLAST finding more true positives for *k<20*, as shown in Fig. 3b. The average difference between BLAST and CB-Search-GL in this range was approximately 3.3%. Afterward, the performance of BLAST declines, falling below the results of the other methods. On the other hand, the results from keratin type II head domains indicated significant differences. In this case, all of the CB-Search variants performed outperformed BLAST, suggesting that this drop in performance is caused by the heuristics used in this method rather than by the alignment algorithm.

**Fig. 4:**
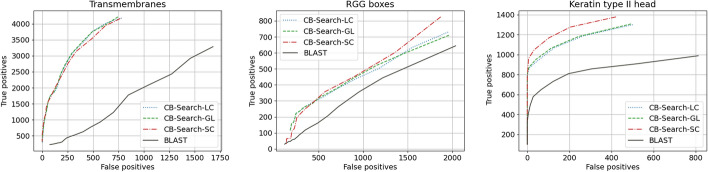
The similarity score used by CB-Search selects significantly more results relevant to the queries in both analyzed cases. The charts display the number of true positives in relation to the number of true negatives for the transmembrane domains, RGG boxes, and keratin type II head

After switching from the limit threshold to the similarity score and E-value for CB-Search and BLAST, respectively, the results diverged significantly, as shown in Fig. 4. In this scenario, CB-Search consistently identified more true positives than BLAST did for all domains. For *4949 ± 16* transmembrane target sequences, which is the maximal value presented in the charts for each method, BLAST revealed only approximately 66.2% true positives, whereas those of CB-Search-GL reached 85.0%, those of CB-Search-SC reached 84.4%, and those of CB-Search-LC were 84.3%. Moreover, for *873 ± 30* target sequences, the BLAST results consisted of only approximately 62.1% true positives, whereas those of CB-Search-GL accounted for approximately 98.0%, those of CB-Search-LC accounted for approximately 98.3%, and those of CB-Search-SC accounted for approximately 98.4%. With respect to the RGG boxes, the differences in the results were smaller, but the metric used in CB-Search still yielded more true positives than the E-value did. With respect to *2683 ± 11* target sequences, BLAST revealed nearly 24.0% true positives, CB-Search-GL 26.6% true positives, CB-Search-LC 27.2% true positives, and CB-Search-SC 30.6% true positives. A greater difference was observed with closer similarity, where for *459 ± 10* target sequences, BLAST revealed only approximately 25.5% true positives, whereas CB-Search-GL identified 47.4% true positives, CB-Search-LC identified 47.1% true positives, and CB-Search-SC identified 43.2% true positives. In the case of the keratin type II head domain, CB-Search again significantly outperformed BLAST. For *102 ± 3* target sequences, all of the methods found sequences containing the query domain. However, for 1800 target sequences, BLAST identified only 54.9% of true positives, while CB-Search found 72.2%, 72.6 and 76.6% of true positives for CB-Search-LC, CB-Search-GL, and CB-Search-SC, respectively.

### Example - K/DE motif search

**Table 1:** The presented parameter sets were used for the similarity analysis of the K-DE motif

	CB-Search-SC	CB-Search-GL	CB-Search-LC	BLAST
Gap open	6	6	6	7
Gap extend	0.5	0.5	0.5	2
Word size	N/A	N/A	N/A	4
Similarity threshold	0.65	0.65	0.65	N/A
Relative threshold	0.1	N/A	N/A	N/A

**Fig. 5:**
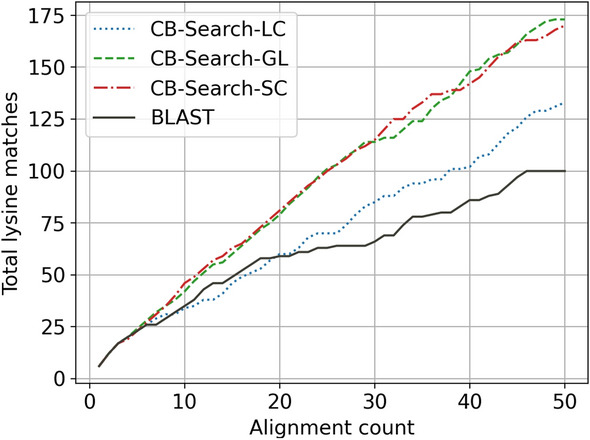
For BLAST, the number of aligned lysines is greater for closer similarities than for more distant similarities, whereas for the CB-Search method, the number of matching lysines increased steadily. The chart illustrates the cumulative sum of matched lysines in the top 50 alignments

**Fig. 6:**
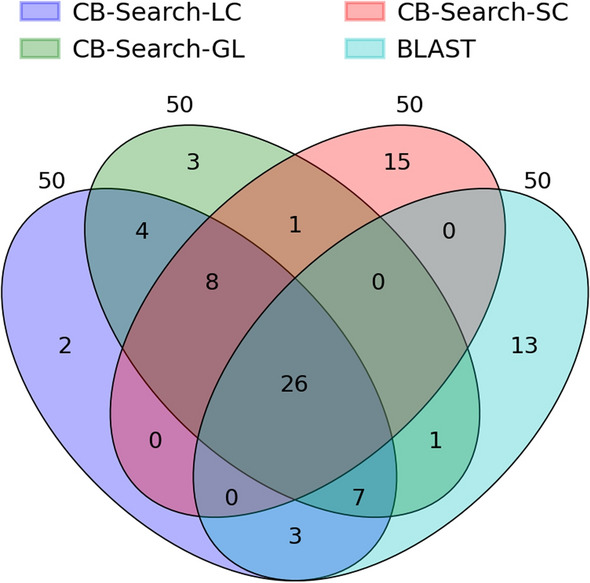
The CB-Search-LC and CB-Search-GL results strongly overlap, whereas CB-Search-SC and BLAST have many unique similarities. The Venn diagram illustrates the overlap of proteins found by each method when the K-DE motif of DNA-directed RNA polymerase delta was searched for similarities

Global and global–local alignment algorithms included all parts of the K-DE motif of DNA-directed RNA polymerase subunit delta in the results more often than local alignment did. Table 1 presents the optimized parameters for the methods. Using these parameters, CB-Search-GL aligned 174 lysines in the first 50 alignments, CB-Search-SC aligned 168 lysines, CB-Search-LC aligned 134, and BLAST aligned only 100 lysines. Their cumulative sum is presented in Fig. 5, which indicates that BLAST revealed fewer lysine matches with distant similarity. The plot for glutamic and aspartic acids is shown in Supplementary Figure S1 and indicates that all of the methods were comparable. Figure 6 presents the overlap of proteins similar to the K-DE motif identified by each method. We noticed that compared with CB-Search-GL and CB-Search-LC, BLAST and CB-Search-SC revealed 15 and 14 unique proteins, respectively, which are high values; on the other hand, 46 proteins in common out of 50 were identified. The 4 results obtained with CB-Search-GL, which were missing from CB-Search-LC, aligned to 10 lysines, whereas CB-Search-LC aligned to only 1 lysine in the records absent from the CB-Search-GL results. Therefore, compared with local alignment, the global–local algorithm identified more similar sequences with lysines at the N-terminus. Interestingly, the CB-Search-SC and CB-Search-GL variants, which align a high number of lysines, were found in as many as 16 nonoverlapping proteins. This suggests that these two variants used simultaneously may reveal more relevant similarities.

### 2-mer filtering performance

**Fig. 7:**
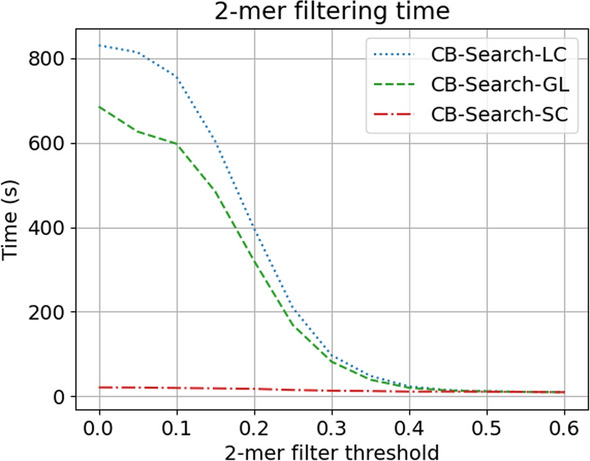
The computation time significantly dropped for threshold value 0.4 for CB-Search-LC and CB-Search-GL. The chart shows relationship between calculation time and filtering threshold value

Using transmembrane domains, we found that 2-mer filtering significantly accelerated calculations with the CB-Search method. The execution times for multiple filter thresholds are presented in Fig. 7. The results indicate that 2-mer filtering reduced computation when changing threashold from 0.0 to 0.3 by 8.6 times for local alignment, 8.4 times for global–local alignment, and only 1.6 times for global alignment with SimiComp. Nevertheless, CB-Search-SC was faster than CB-Search-GL and CB-Search-LC were, as SimiComp in the first variant already selects relevant fragments for alignment. Consequently, these results also highlight the high computational efficiency of SimiComp.

**Fig. 8:**
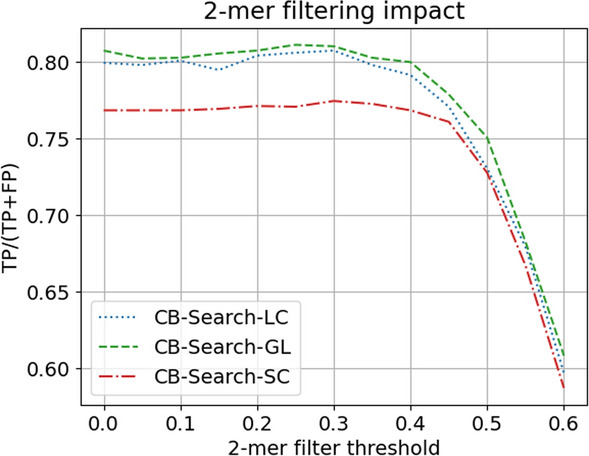
The 2-mer filter slightly increased true positive fraction at a threshold of 0.3. However, as the threshold increased the value above 0.4 the number of transmembrane domains in the results decreased significantly. the y-axix represents the fraction of transmembrane domains within top 50 results, while the x-axis is 2-mer filtering threshold

We found that this acceleration is not linked to a drop in accuracy when searching for similar transmembrane domains. The analysed domains are challenging for such filtering solutions, as they could evolve independently; consequently, residues in two similar sequences may be positionally independent. Figure 8 shows the ratio of true positives to false positives in the top 50 results obtained using optimised parameters while changing 2-mer threshold from 0.0 to 0.6.

**Fig. 9:**
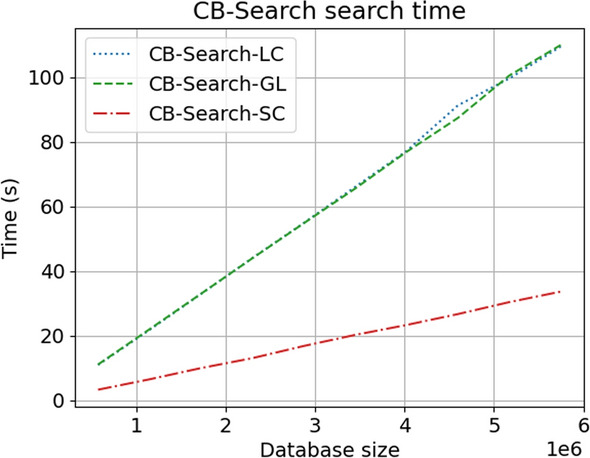
The execution time of CB-Search depends linearly on the database size. However, the SimiComp algorithm in CB-Search-SC accelerates this process, as alignments are only computed for identified fragments. The experiment utilized a single core on an Intel(R) Xeon(R) CPU E5-4667 v4 @ 2.20 GHz

To evaluate scalability relative to database size, we repeatedly executed CB-Search using the K-DE motif of the DNA-directed RNA polymerase subunit delta as a query against databases of various sizes. As shown in Fig. 9, CB-Search-LC and CB-Search-GL exhibit comparable execution times, whereas CB-Search-SC acheves a significantly lowerer runtime. This performance gain in CB-Search-SC is attributed by SimiComp, which restricts the alignment algorithm to evaluate only relevant fragments of the database sequences; in contrast, both CB-Search-LC and CB-Search-GL require aligning full sequences.

## Discussion

In this paper, we introduced the CB-Search method, which is designed to search for similar motifs with biased compositions. This method offers three alignment approaches: an algorithm for identifying fragments with similar compositions, 2-mer filtering, and the similarity score metric. In the analyses, we utilized CB-Search in three variants: CB-Search-LC which uses the local alignment algorithm; CB-Search-GL, which uses global–local alignment; and CB-Search-SC, which uses global alignment with SimiComp. These variants, were compared with BLAST in the similarity analyses of transmembrane domains and RGG boxes. Additionally, we evaluated the quality of the alignments when we searched for similarities to the K-DE motif of the DNA-directed RNA polymerase subunit delta by examining the inclusion of all the query sequence submotifs.

The selection of an appropriate alignment algorithm for motifs with biased compositions remains an unresolved issue. In this research, we examined how different alignment algorithms influence the number of true positives in the results for transmembrane domains, RGG boxes, and keratin type II head. For these three domains, we obtained mixed results. For transmembrane domains, the global–local alignment algorithm yielded more true positives for most of the limits investigated, whereas for RGG boxes, the BLAST implementation of local alignment performed better for close similarity. On the other, for kerating type II head all CB-Search variants outperformed BLSAT, suggesting that in this case failed heuristics used in BLAST. These findings are inconsistent with those published for standard composition domains, where global–local alignment demonstrated results superior to those of other alignment strategies [51]. This suggests that motifs with biased compositions require more careful consideration when selecting an alignment algorithm. Currently, we recommend evaluating the true positive content of available algorithms whenever possible. However, some guidance can be derived from existing knowledge for situations where this is not feasible. There are known domains for which length is critical for specific functions [52]. In such cases, it seems logical to use global alignment with SimiComp, which penalizes length differences. Conversely, local alignment may be more suitable for queries in which functional domain boundaries are difficult to define. In other scenarios, global–local alignment may be appropriate when a query sequence contains multiple submotifs. Therefore, information about the length variability of domains would be valuable in databases.

The similarity score used in CB-Search is more relevant to evaluate alignments of motifs with biased compositions than the E-value. The results demonstrated that biased residues in sequence comparisons may need to be emphasized in scoring matrices to ensure proper alignment. This operation, however, can lead to a positive expected score of a scoring matrix, which disqualifies the bit score and E-value [43, 53]. E-value is specifically designed for homologous proteins, as it estimates how often a similar sequence might be found in a particular database by chance [8]. However, this estimation becomes irrelevant for low-complexity regions, where similar nonhomologous sequences may frequently appear in databases [7]. The presented similarity score addresses these issues by normalizing alignment scores calculated for motifs of different compositional classes (e.g. A-rich and W-rich) and by being independent of the lambda parameter. Additionally, as presented in Fig. 4, when the similarity score is set as a threshold, many more motifs that are relevant to a query than the E-value are selected.

The relationship between similarity scores and scoring matrices influences the alignment of compositionally biased motifs and warrants further research. While similarity scores normalize alignments across different classes of compositionally biased motifs, it has still significant impact on motifs composed of multiple biased residues. In collagen, for instance, glycine is essential for triple-helix formation and arguably requires a higher diagonal score than proline, despite proline’s role in structural stabilization [54]. We hypothesize that this issue could be addressed by designing collagen-specific scoring matrices or by pre-adjusting a uniform-diagonal matrix to emphasize frequently occurring residues, thereby appropriately elevating glycine’s score. Conversely, standard matrices like BLOSUM62 assign a higher match score to proline than glycine, and the difference is further amplified by default BLAST adjustments. Therefore, improving scoring matrices and their adjustment algorithms for biased motifs remains a key objective for future research [43]. Until these generalized solutions are refined, the application of specialized scoring matrices is recommended for compositionally biased motifs [55].

Searching methods are widely utilized in bioinformatics and can be used in biological analyses, automated annotation, and clustering methods. To date, scientists developed specialized tools for specific domains and regions including HEAT repeats, intrinsically disordered regions and transmembrane domains [39, 40, 56]. Nevertheless, many domains still lack dedicated tools; researchers would therefore benefit from general purpose methods designed for motifs with biased compositions. Furthermore, such general purpose tools could help organize existing knowledge by providing a comprehensive overview of the relationships between these motifs. This framework, could be then refined by improving these tools or by using specialized tools for specific domains. In this study, we demonstrated that our method can increase the number of true positives in the results and find hits containing all the query sequence features. Therefore, the method could be applied in biological analyses investigating transmembranes or RGG boxes that utilize search methods allowing for more precise results. It may also be beneficial in protein clustering methods that rely on similarity distances between sequences [57]. Another application of our method is the prediction of functional regions in proteins through protein similarity search techniques [58, 59]. In these cases, the challenge of search algorithm performance varying across different motifs can be effectively addressed using ensemble methods [60].

## Conclusions

We have developed CB-Search, which is specifically designed for searching for similar motifs with biased compositions. Our new similarity score metric outperforms the state-of-the-art E-value metric in terms of the fraction of true positives when analyzing transmembrane domains, RGG boxes, and keratin type II head. The introduction of 2-mer filtering significantly reduced the search time by eliminating sequences that lacked potentially similar motifs. These results encourage taking this method into account for a wide range of analyzes and new approaches utilizing motifs with biased compositions, which often consist of multiple types of submotifs. Local alignment-based methods may omit some parts of the query sequence, whereas global–local and global alignment with our SimiComp method, includes all the query components for comparison. Therefore, selecting an appropriate alignment algorithm is crucial for efficiently searching for similarities between motifs with biased compositions. In this research, we also provide guidance and hypotheses on how to choose the most suitable algorithm for different classes of problems.

## Additional file

Supplementary file 1 (pdf 253 KB)

## Data Availability

The datasets analysed during the current study were retrieved from the UniProt database at https://ftp.uniprot.org/pub/databases/uniprot/previous_releases/release-2023_03/ and InterPro at https://ftp.ebi.ac.uk/pub/databases/interpro/releases/103.0/. LCRAnnotationsDB and scripts used in this study are available in the Zenodo repository at https://doi.org/10.5281/zenodo.19472567. The code used in this study is available in the GitHub repository at https://github.com/patryk-jarnot/cb-search.
