# Supplementary material for: CB-Search: a method for searching for similar protein motifs with biased compositions
Source: BMC Bioinformatics. 2026 Jun 4;27:173. doi: 10.1186/s12859-026-06509-w (PMC13466491; doi:10.1186/s12859-026-06509-w)
Supplement: Supplementary file 1 [file 12859_2026_6509_MOESM1_ESM.pdf]

CB-Search: A method for searching for similar protein motifs with biased compositions – supplementary materials

Patryk Jarnot[1,*]

[1]Department of Computer Networks and Systems, Silesian University of Technology
[*]patryk.jarnot@polsl.pl

Global-local alignment

This algorithm aligns a query sequence globally to a database sequence locally, emphasizing the presence of all query sequence features in local similarities. It can be considered a subtype of semi-global alignment with affine gap penalties that is penalty free only in a query sequence at both ends. However, the semi-global alignment is badly defined and it is frequently described as an alignment in which one end of a sequence is opened for gap penalties while the other end is fixed overlapping one end of another sequence. Therefore, we defined the configuration used in this paper as global-local alignment [?, ?]. Formally, the method first constructs gap affinity matrices from both sequences using Equation 1 [?].

$$\begin{aligned}
S(i,j) &= \begin{cases} \infty & (i=0, j\neq 0) \\ 0 & (j=0) \\ \max\begin{cases} S(i-1,j-1)+s(x_i,y_j) \\ I_x(i-1,j-1)+s(x_i,y_j) \\ I_y(i-1,j-1)+s(x_i,y_j) \end{cases} & (i\neq 0, j\neq 0) \end{cases} \\
I_x(i,j) &= \begin{cases} \infty & (i=0) \\ -d-je & (i\neq 0, j=0) \\ \max\begin{cases} S(i-1,j)-d \\ I_x(i-1,j)-e \end{cases} & (i\neq 0, j\neq 0) \end{cases} \\
I_y(i,j) &= \begin{cases} -d-je & (i=0, j\neq 0) \\ \infty & (j=0) \\ \max\begin{cases} S(i,j-1)-d \\ I_y(i,j-1)-e \end{cases} & (i\neq 0, j\neq 0) \end{cases}
\end{aligned} \tag{1}$$

where d is the gap open penalty, e is the gap extend penalty and $s(x_i, y_j)$ is a value of a scoring matrix for amino acids x_i and y_j. The algorithm then traceback from a cell with a maximal value in the last column ($j = N$) of the matrices and continues it until $j = 0$.

Searching for compositionally biased domains using MMseqs

We attempted to optimize MMseqs parameters as this method is widely applied for sequence similarity searches, and could be used to evaluate CB-Search performance, but unfortunately it found unexpectedly low number of similar domains with biased compositions. First of all, we set comp-bias-correction and mask parameters to 0 in order to allow for searching for sequences with biased compositions as it is recommended by the documentation. To avoid also other filtering which could filter out sequences containing compositionally biased regions, we disabled prefiltering by setting option prefilter-mode to 2. E-value we set to 1000000, which is very high. Then, we used gridsearch to optimize gap open and gap extend penalties to increase the number of true positives. Gap open penalty, we optimized from 9 to 13 in increments of 1, while gap extend penalty by checking values 1 and 2. Despite this effort, we obtain unsatisfactory results. Figure S1 shows that for transmembranes we obtained just few true positives. Their fraction slightly changed when tweaking gap extend penalty, but the results were insensitive to gap open penalty. For RGG boxes, the method found no true positives at all. Therefore, this method was excluded from the comparison analysis.

Since the optimization analysis showed unexpectedly poor performance of MMseqs, we also systemically tweaked the rest of the parameter to reject the possibility of misunderstaning their meaning. As base parameters, we used the parameters from the optimization analysis with default values of gap open and gap extend penalties. For the analysis, we randomly picked two transmembrane domains from fms-related tyrosine kinase 3 ligand (P49771) at position 183-201, and palmitoyltransferase app (A2VEY9) at position 348-366. Then, we tested the following options by adding only one to the above parameters and checked whether they increase the number of results: s 1, s 4, s 7.5, k 1, k 4, k 8, target-search-mode 0, target-search-mode 1, mask-prob 0, mask-prob 0.5, mask-prob 1, min-ungapped-score 0, min-ungapped-score 10, min-ungapped-score 30, spaced-kmer-mode 0, spaced-kmer-mode 1, alignment-mode 0, alignment-mode 1, alignment-mode 2, alignment-mode 3, alignment-mode 4, seq-id-mode 0, seq-id-mode 1, seq-id-mode 2, corr-score-weight 0, corr-score-weight 0.5, corr-score-weight 1, zdrop 20, zdrop 60, zdrop 80, mask-profile 0, mask-profile 1, e-profile

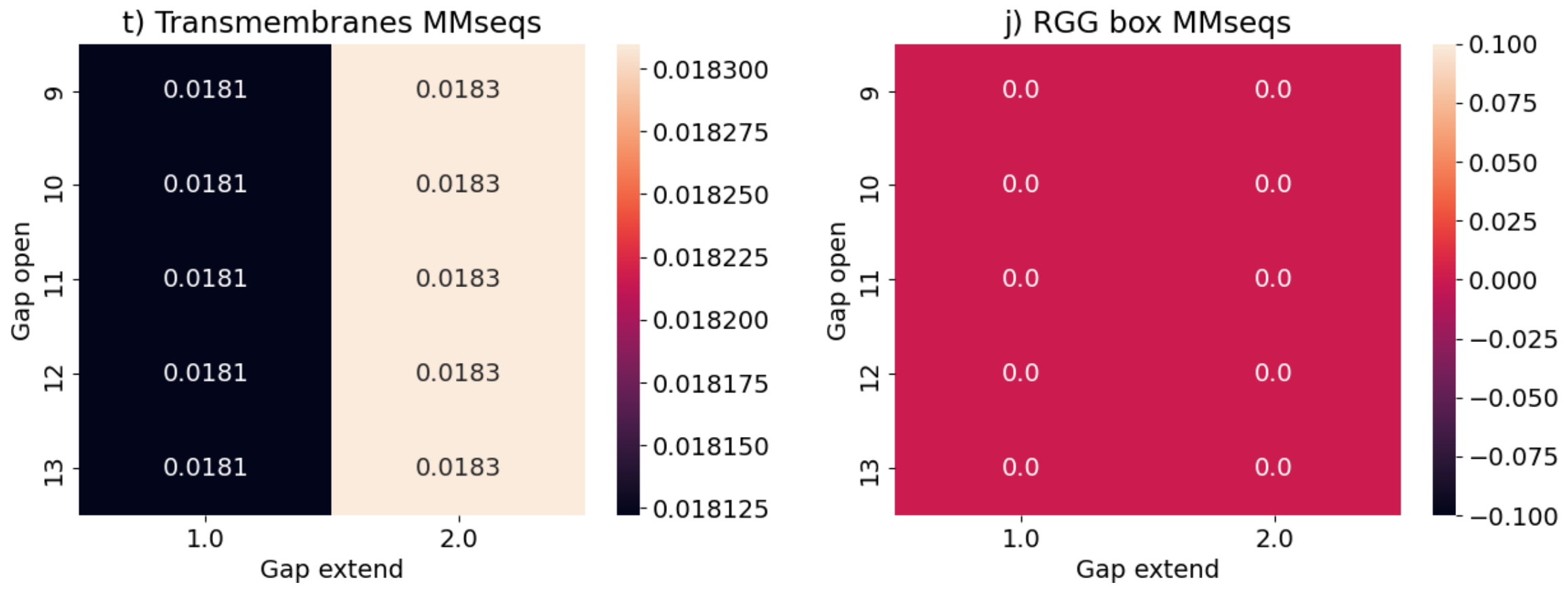

Figure 1: Heatmaps showing gap open and gap extend parameter optimization with respect to the number of true positives.

0.1, e-profile 0.0001, e-profile 1e-06, wg 0, wg 1, filter-msa 0, filter-msa 1, filter-min-enable 10, filter-min-enable 100, filter-min-enable 1000, max-seq-id 0.0, max-seq-id 0.5, max-seq-id 1.0, qid 0.0, qid 0.5, qid 1.0, qsc -50.0, qsc 25.0, qsc 100.0, cov 0.0, cov 0.5, cov 1.0, diff 10, diff 100, diff 10000, pseudo-cnt-mode 0, pseudo-cnt-mode 1, num-iterations 2, num-iterations 3, num-iterations 4, rescore-mode 1, rescore-mode 2, rescore-mode 3, rescore-mode 4, allow-deletion 1, min-length 1, min-length 10, min-length 100, max-length 100, max-length 200, max-length 300, search-type 1, start-sens 1.0, start-sens 2.0, start-sens 8.0, overlap 0.0, overlap 0.5, overlap 1.0. For fms-related tyrosine kinase 3 ligand, none of the parameters increased even the number of results and there were at most only 14 alignments. For palmitoyltransferase app it was similar with maximal number of 7 alignments.

K-DE motif from RNA polymerase

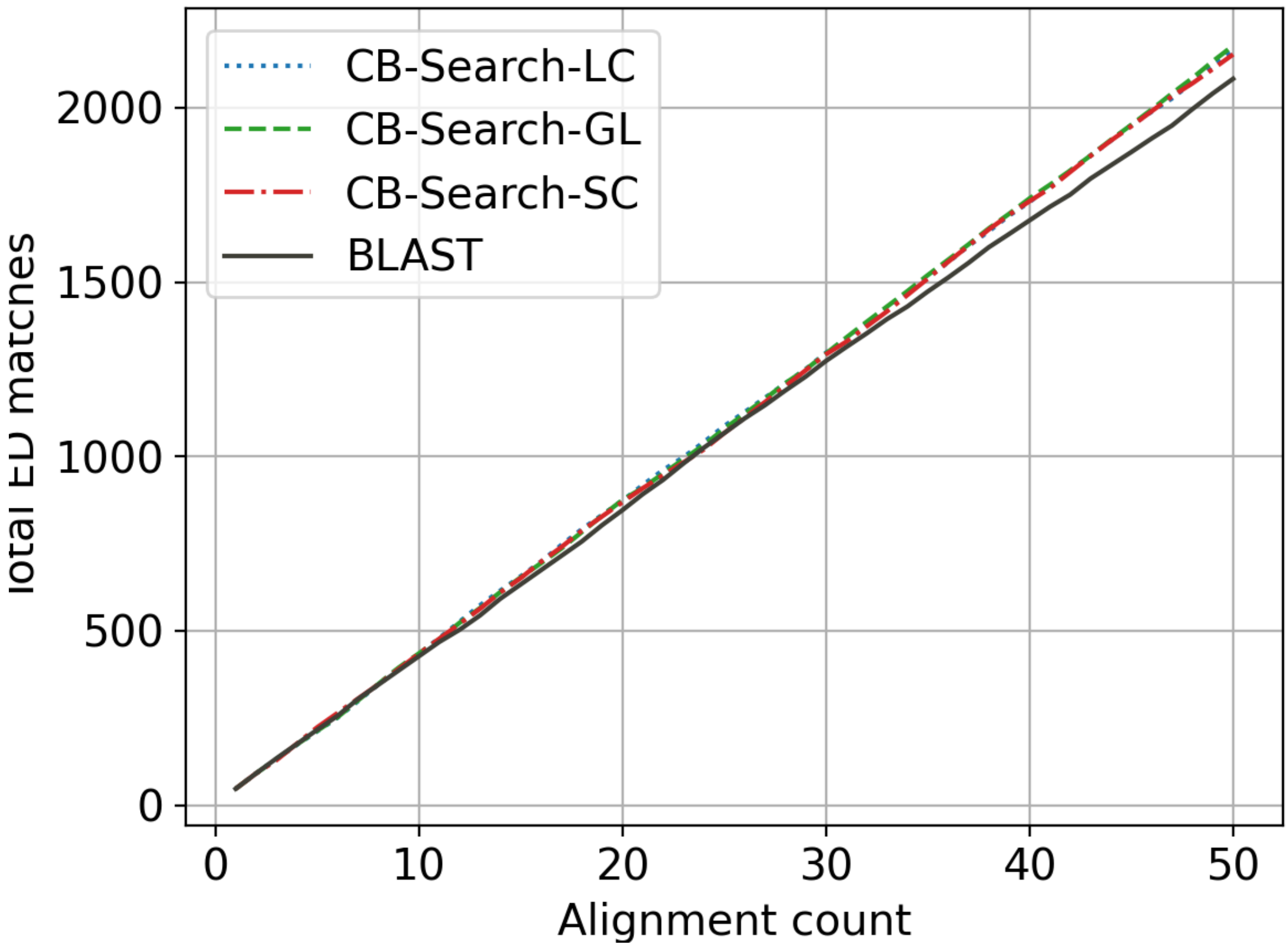

Figure 2: Cumultive sum of glutamic and aspartic acids matches in top 50 alignments of DNA-directed RNA polymerase subunit delta. Matches of glutamic acid to aspartic acid and vice versa were also counted considering these residues as similar.
